# Quantitative Kinetic Analysis of Hydraulic Aging in EPDM Rubber: Evolution of Functional Properties

**DOI:** 10.3390/polym18131604

**Published:** 2026-06-28

**Authors:** Djaffar Bouguedad, Dahmane Mouri, Aomar Hadjadj

**Affiliations:** 1 Laboratoire de Génie Electrique, Université Mouloud Mammeri, Tizi-Ouzou 15000, Algeria; dahmane.mouri@ummto.dz; 2 Matériaux & Ingénierie Mécanique, Université de Reims Champagne-Ardenne, 51100 Reims, France

**Keywords:** EPDM, hydraulic aging, water diffusion, mechanical properties, electrical properties

## Abstract

The long-term effects of water immersion on the physicochemical and functional properties of ethylene-propylene-diene monomer (EPDM) elastomer, widely used as insulation in medium-voltage electrical cables, were investigated over a period of 140 days at room temperature. A multi-scale experimental approach combining complementary characterization techniques was employed to establish quantitative correlations between moisture-induced physicochemical changes and the resulting evolution of functional performance. Water uptake, governed by Fickian diffusion kinetics, remained limited to 0.30 wt%. At the surface, progressive roughening was observed alongside the formation of microcavities and microcracks. Leaching of mineral fillers and an increase in surface polarity were found to enhance wettability. These combined physicochemical alterations translated into measurable degradation of functional properties, with two distinct kinetic regimes identified. Shore hardness, volume resistivity, and dielectric strength underwent rapid deterioration within the first few days of immersion, whereas tensile strength, elongation at break, dielectric permittivity, and dielectric loss factor evolved more gradually over timescales of several tens of days. Temporal profiles for each property were fitted to appropriate models, and characteristic degradation timescales were estimated. These findings provide a structured, physically grounded picture of EPDM degradation under water exposure and offer quantitative data to support the development of service-life prediction models for cable insulation systems.

## 1. Introduction

The saturated hydrocarbon backbone of EPDM confers exceptional resistance to heat, weathering, ozone, UV radiation, chemical attack, and mechanical deformation, making it one of the most extensively studied and industrially deployed elastomers [1]. Its versatility across demanding sectors, including nuclear, aerospace, automotive, construction, energy, electronics, and battery systems, stems from this inherent stability, which can be further tailored through the incorporation of fillers and additives to optimize mechanical, dielectric, and thermal performance [2]. In medium-voltage (MV) and high-voltage (HV) cable insulation, EPDM is routinely compounded with inorganic fillers such as alumina trihydrate (ATH), silica, or magnesium hydroxide to enhance dielectric performance, thermal stability, and fire resistance [3,4]. The resulting compounds typically exhibit volume resistivities on the order of 10^15^ Ω.cm and dielectric strengths exceeding 20 kV/mm [5]. Over a nominal service life of 20 to 40 years, however, these properties are not static. Indeed, the insulation is subjected to a range of environmental and operational (thermal, photochemical, chemical, electrical, and hygroscopic) stresses that progressively alter its physicochemical structure and degrade its functional performance [6].

The aging behavior of EPDM under individual stress factors has been studied extensively. Thermo-oxidative aging initially increases hardness and tensile strength before oxidative chain scission reduces elongation at break and promotes embrittlement [7]. UV exposure induces surface discoloration, modifies surface energy, and generates oxygenated functional groups (C–O and C=O), initiating a degradation front that propagates inward from the surface [8]. Exposure to aggressive solvents or alcohols triggers oxidative chain degradation, with consequent loss of mechanical integrity [9]. Electrical stresses associated with high-field polarization have been linked to the gradual deterioration of insulating properties and the onset of space charge accumulation [10].

Even under outdoor weathering conditions, for which EPDM is reputed to offer service lives exceeding 50 years in temperate climates, some electrical and mechanical properties can be measurably affected after only a few months of exposure, while other bulk properties remain comparatively unaffected [11]. Despite this body of work, aging induced by water exposure has received considerably less attention, despite its direct relevance to a large fraction of installed cable infrastructure. Underground distribution cables are routinely buried in moist or saturated soils, installed in flooded conduits, or terminated in environments exposed to rainwater ingress; submarine cables operate under continuous seawater immersion. In these conditions, water penetration can act synergistically with hydrolytic and oxidative mechanisms, producing micro-cracking, swelling, or plasticization depending on the chemistry, temperature, and duration of exposure. The limited studies addressing hygroscopic aging tend to combine water exposure with concurrent stresses (electrical, thermal, or photochemical), making it difficult to isolate the specific contribution of moisture. The available data suggest that water absorption promotes the formation of dipolar species and the leaching of mineral fillers, while also inducing matrix oxidation and chain scission. These combined degradation mechanisms result in a significant deterioration of both electrical and mechanical properties.

The present study addresses this gap through a systematic, multi-scale investigation of EPDM aging under controlled hydrolytic conditions. Samples were fully immersed in water at room temperature for 140 days, and their evolution was tracked using an integrated suite of electrical, mechanical, and physicochemical characterization techniques. A central objective was to resolve and quantify the two distinct (rapid and gradual) kinetic regimes observed in the degradation of functional properties and to determine their respective characteristic timescales. The outcomes are intended to deepen the fundamental understanding of moisture-induced aging in filled EPDM systems and to provide quantitative data supporting the development of service-life prediction models and the optimization of insulation formulations for next-generation cable systems.

## 2. Materials and Methods

### 2.1. Material

The insulating compound investigated in this study was supplied by CABEL (Algiers, Algeria) in the form of vulcanized elastomeric sheets. Its formulation is based on EPDM pellets (Nordel 2744, Dow Chemical, manufactured by Du Pont Dow Elastomers S.A., Horgen, Switzerland) as the primary polymer matrix, reinforced with two mineral fillers: precipitated calcium carbonate (Chalk EV) and anhydrous aluminosilicate (Whitetex Chalk). A paraffinic mineral oil (Torada S22) was incorporated as a plasticizer to improve processability and flexibility, while paraffin wax was added as an internal lubricant to facilitate mixing and release. Interfacial adhesion between the elastomeric matrix and the inorganic fillers was promoted using a β-methoxyethoxy silane coupling agent. Vulcanization was carried out using dicumyl peroxide (Perkadox BC) as the crosslinking initiator, in conjunction with triallyl cyanurate (TAC) as a co-agent to increase crosslink density and improve network homogeneity. Long-term thermal and oxidative stability was ensured by a binary antioxidant system comprising a trimethyl dihydroquinoline polymer (Flectol H/Permanax TQ) and the zinc salt of 2-mercaptobenzimidazole (Vulcanox ZMB2).

Compounding was performed in an internal mixer at a controlled temperature of 80 °C for 30 min to ensure thorough and homogeneous dispersion of all constituents within the elastomeric matrix. The vulcanizing agents were introduced in a second step, once sufficient homogeneity had been confirmed, to prevent premature crosslinking during mixing. The resulting compound was then discharged and cooled to ambient temperature on a clean planar surface. Sheets were subsequently shaped and vulcanized simultaneously in a hydraulic compression molding press at 180 °C under an applied force of 300 kN for 10 min. This process yielded square elastomeric sheets of 25 × 25 cm^2^, with thicknesses of 1 or 2 mm depending on the characterization technique requirements.

### 2.2. Hydrolytic Aging Procedure

Hydrolytic aging experiments were conducted in accordance with ISO 1817 [12]. Elastomeric specimens were fully immersed in domestic water contained in sealed glass vessels at a temperature of 22 °C. Samples were withdrawn at regular intervals of 14 days over a total immersion period of 140 days. Upon retrieval, each specimen was gently blotted with dry absorbent paper to remove surface moisture before characterization. As samples were cut into pieces of lateral size larger than the thickness, the diffusion can be considered to only occur in the thickness of the sample. The very mild aging conditions used in this study (immersion in tap water at room temperature) attempted to replicate the actual operating conditions of an underground cable exposed to water as closely as possible. The tap water used was of average hardness, with a mineral concentration of around 300 mg/L (including 40 mg/L of Ca, 20 mg/L of Mg and 170 mg/L of bicarbonates) and a conductivity of around 300 µS/cm. The water was not replaced throughout the immersion period, but the temperature was maintained constant.

### 2.3. Characterization Techniques

Specimens were then subjected to a comprehensive battery of electrical, mechanical, and physicochemical analyses. Five nominally identical samples were used to determine a mean value and the corresponding error bar for each aging condition and for most of the measurements performed.

#### 2.3.1. Surface Morphology and Chemical Composition

The surface morphology of aged and unaged specimens was examined by scanning electron microscopy (SEM) using a ZEISS EVO 15 environmental SEM (Zeiss, Oberkochen, Germany) equipped with an energy-dispersive X-ray spectroscopy (EDX) system, enabling simultaneous elemental analysis. Surface wettability was assessed by static contact angle measurements using a Krüss DSA 30 goniometer (KRÜSS GmbH, Hamburg, Germany). Before each measurement, specimens were cleaned sequentially in ultrasonic baths of acetone and deionized water to remove surface contaminants. A 2 µL droplet of deionized water was deposited on the sample surface, and the contact angle was determined by the sessile drop method.

#### 2.3.2. Water Uptake

Gravimetric water absorption was monitored in accordance with ASTM D471 [13]. Prior to immersion, unaged samples were dried to constant mass and weighed using a Sartorius CP 225D (Sartorius, Göttingen, Germany) analytical balance (0.1 mg of resolution). At each aging interval, five specimens were retrieved from the immersion medium, carefully blotted dry with absorbent paper, weighed immediately, and returned to the aging environment to resume exposure. Water uptake at a time *t* was expressed as the percentage mass gain ΔM(*t*) relative to the initial dry mass.

#### 2.3.3. Chemical and Structural Analysis

Chemical changes were monitored by attenuated total reflectance Fourier-transform infrared (ATR-FTIR) spectroscopy using a IRAffinity-1S FTIR spectrometer (Shimadzu Corporation, Kyoto, Japan) over the 400–4000 cm^−1^ spectral range, with a resolution of 4 cm^−1^ and signal averaging over 32 scans to ensure an adequate signal-to-noise ratio. Crystalline structure was investigated by X-ray diffraction (XRD) using a Malvern Panalytical Empyrean diffractometer (Malvern Panalytical, Almelo, The Netherlands) with a copper anode emitting Cu Kα_1_ (λ = 1.540598 Å) and Cu Kα_2_ (λ = 1.544426 Å) radiation. Diffraction patterns were collected over the 5–80° 2θ range with a step size of 0.026° and a counting time of 0.5 s per step. Thermal stability was assessed by simultaneous thermogravimetric analysis and differential scanning calorimetry (TGA/DSC) using a Netzsch STA 449 F Jupiter instrument (Netzsch, Selb, Germany). Specimens of approximately 10 mg were heated from 25 to 1000 °C at a rate of 10 °C·min^−1^ under a nitrogen purge flow of 50 mL/min.

#### 2.3.4. Mechanical Properties

Uniaxial tensile tests were performed on dumbbell-shaped specimens (ASTM D638, Type I) using a Zwick/Roell Z050 universal testing machine (ZwickRoell, Ulm, Germany) at a crosshead speed of 50 mm.min^−1^ and room temperature. Tensile strength and elongation at break were determined from the resulting stress–strain curves [14]. Shore A hardness was measured with a Zwick/Roell HPE digital durometer (ZwickRoell, Ulm, Germany) equipped with a stainless-steel indenter and an integrated displacement sensor, in accordance with ISO 868 [15].

#### 2.3.5. Electrical and Dielectric Properties

Volume resistivity was determined using a high-precision teraohmmeter (Knick, Berlin, Germany) fitted with a guarded three-electrode system to suppress surface leakage currents. A DC voltage of 500 V was applied for an electrification time of 60 s prior to measurement, in accordance with standard protocols for insulating materials [16]. Dielectric strength was evaluated at 50 Hz using BAUR spintermeters (DTA 100 and PGO 90A, BAUR GmbH, Sulz, Austria) with a voltage ramp of 2 kV.s^−1^. Tests were carried out at room temperature with specimens immersed in agitated insulating oil to promote volume breakdown under quasi-uniform field conditions. Dielectric strength was calculated as the ratio of breakdown voltage to specimen thickness [17]. The relative permittivity and dielectric loss factor (tanδ) were measured at the material’s rated service temperature of 90 °C using a Tettex AG Schering bridge (Tettex AG, Basel, Switzerland), under a sinusoidal excitation voltage of 2 kV at 50 Hz. Circular specimens of 75 mm diameter were used to ensure adequate electrode coverage and field uniformity [18].

## 3. Results and Discussion

### 3.1. Microstructural and Morphological Changes

**Figure 1:**
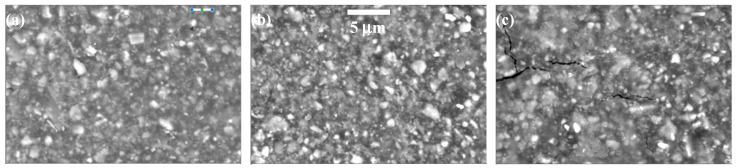
SEM images of unaged (**a**), 70 days aged (**b**) and 140 days aged (**c**) EPDM samples.

SEM images of EPDM specimens before and after immersion are presented in Figure 1. In the unaged state, the surface appears dense and homogeneous, with no evidence of cracking, cavitation, or interfacial delamination. The moderate surface roughness observed is characteristic of compression-molded EPDM and reflects the imprint of the mold surface and the viscoelastic relaxation of the compound during processing [19]. After 70 days of immersion at room temperature, surface alterations become apparent at the microscale, though they remain imperceptible to the naked eye. An increase in surface roughness and the emergence of isolated microcavities are consistent with localized swelling driven by water diffusion into free-volume regions and along polymer/filler interfaces. Although EPDM is generally regarded as weakly polar, recent studies have demonstrated that water can penetrate elastomeric networks through these preferential pathways, inducing localized plasticization and chain relaxation [20,21]. This relaxation reduces surface stiffness and enhances topographical contrast under SEM observation. Concurrently, the partial migration and leaching of low-molecular-weight species, such as plasticizers and processing aids, contribute to the nucleation of micro-voids and the development of a granular surface texture, as commonly reported in elastomers subjected to prolonged aqueous exposure [22,23]. After 140 days, surface degradation is considerably more pronounced. High cavity density, cavity coalescence, and marked surface heterogeneity collectively indicate a transition from reversible hydric swelling to irreversible microstructural damage. This progression is consistent with a two-stage aging mechanism (initial water uptake and swelling, followed by surface destabilization), previously documented in EPDM and analogous elastomers under prolonged immersion [24]. The growth and coalescence of cavities progressively impair interfacial cohesion between the polymer matrix and the inorganic reinforcing fillers, generating mechanically weakened zones and increasing surface porosity [25]. This microstructural evolution is expected to have direct consequences for the bulk mechanical and dielectric properties discussed in subsequent sections.

**Figure 2:**
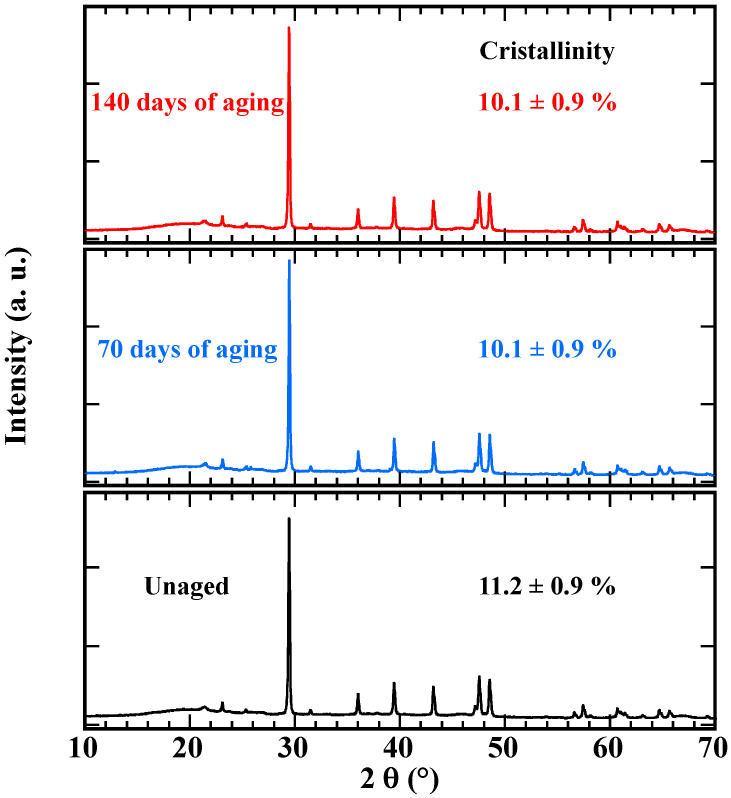
XRD pattern of unaged and aged (70 and 140 days) EPDM samples.

Figure 2 shows a comparison of the XRD patterns of unaged and 70- and 140-day immersed EPDM specimens. These patterns reveal a significant amorphous halo extending from 15° to 28° (area A_am_), alongside a series of crystalline peaks, with the primary peak positioned at 2θ = 21.5° (area A_cryst_). There are no significant differences in peak position, width or relative intensity across the three conditions, indicating that prolonged water exposure at room temperature does not alter the nature or distribution of crystalline domains within the EPDM matrix. The crystalline fraction of the sample can be determined based on the ratio A_cryst_/(A_cryst_ + A_am_) [26]. Quantitative analysis confirms that the crystallinity remained unchanged within the limits of measurement uncertainty, indicating no significant structural reorganization. The crystallinity was 11.2 ± 0.9% in the unaged state and 10.1 ± 0.9% after 140 days of immersion.

This stability is attributable to the inherent resistance of EPDM’s saturated hydrocarbon backbone and crosslinked network to hydrolytic attack, which limits polymer-water interactions under moderate aqueous conditions [27]. Water uptake under these conditions preferentially affects the amorphous regions, where chain mobility is higher and free volume is greater, without disrupting the crystalline domains [27,28,29]. This nearly constant crystallinity observed is therefore consistent with a sorption mechanism confined to the amorphous phase, in agreement with recent reports showing that water transport in EPDM is governed by crosslink density and oxidative state rather than by crystalline reorganization [30].

### 3.2. Chemical and Compositional Changes

**Figure 3:**
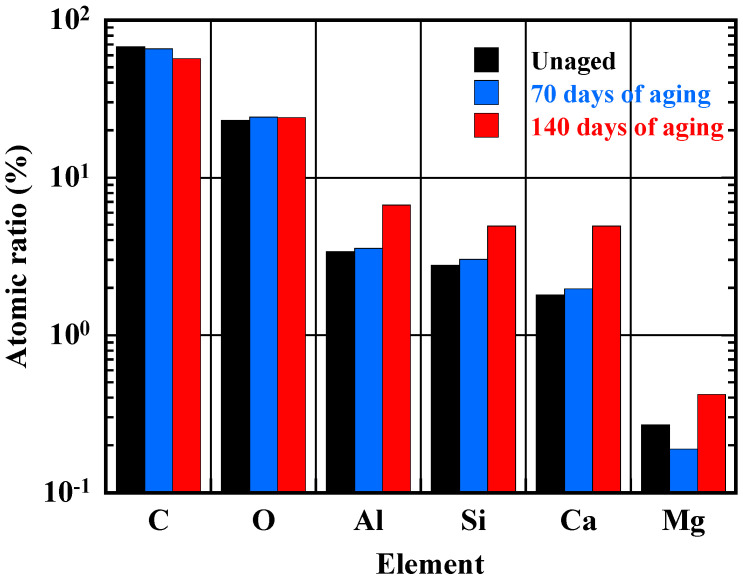
EDX atomic ratio of unaged and aged (70 and 140 days) EPDM samples.

Changes in the surface elemental composition of the filled EPDM elastomer were monitored by energy-dispersive X-ray spectroscopy (EDX) before and after immersion. The corresponding atomic percentages are presented in Figure 3. In the unaged state, the surface composition is dominated by carbon, originating from the EPDM matrix, and by oxygen and inorganic elements (Ca, Al, and Si) associated with the mineral filler system (CaCO_3_ and aluminosilicate phases). The relatively high oxygen content reflects contributions from filler oxides, minor surface oxidation, and the inherent sensitivity of EDX analysis to the oxygen-rich environment of polymeric substrates [31].

After 70 days of immersion, only marginal variations in elemental composition are observed, indicating limited surface oxidation and the absence of significant chemical degradation of the EPDM backbone under mild aqueous conditions at this stage [32]. After 140 days, however, EDX analysis reveals a pronounced decrease in relative carbon content accompanied by an increase in the O/C ratio, suggesting enhanced interaction between water molecules and the polymer backbone. More strikingly, a substantial enrichment in calcium, aluminum, and silicon indicates a transition from a polymer-dominated surface to one increasingly governed by inorganic phases. This compositional shift can be attributed to two concurrent mechanisms: (i) the deposition of mineral species from the aqueous environment, particularly Ca-rich scaling, and (ii) the progressive exposure of intrinsic mineral fillers resulting from superficial erosion or dissolution of the polymer matrix. Taken together, these observations indicate that the aging of filled EPDM under ambient water immersion is primarily driven by surface physicochemical processes, including ionic adsorption, mineral precipitation, and surface reorganization, rather than by bulk chemical degradation [33,34]. The water used is moderately hard. However, as the Ca, Al and Si signals do not increase monotonically with immersion time, the mineral deposition hypothesis seems unlikely. This is particularly evident when considering the SEM images in Figure 1, which do not appear to support this hypothesis. Only experiments using deionised water could definitively resolve this issue.

**Figure 4:**
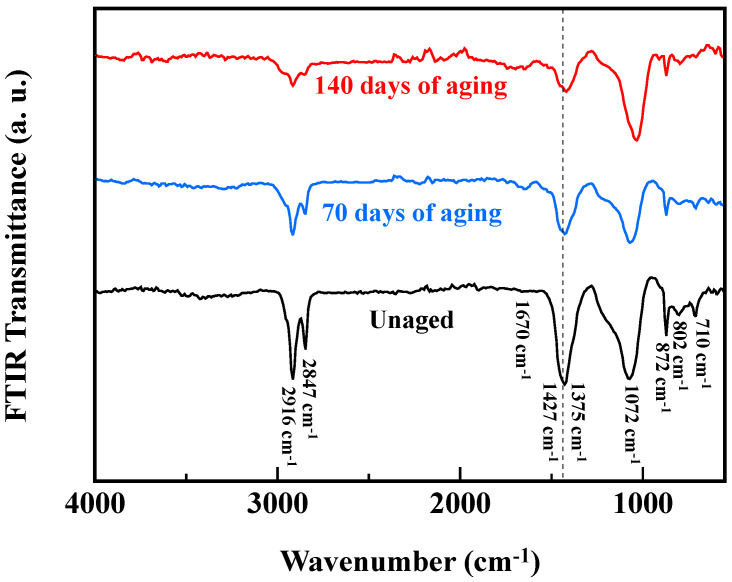
FTIR spectra of unaged and aged (70 and 140 days) EPDM samples. The dotted line is used to highlight the shift in the bending modes of –CH_2_– and –CH_3_ groups.

ATR-FTIR spectra of EPDM specimens before and after immersion are shown in Figure 4. The absorption band near 800 cm^−1^ is typically employed to monitor the consumption of C=C double bonds [35], whereas the band near 1700 cm^−1^ enables the monitoring of C=O carbonyl group formation during the oxidative or thermo-oxidative degradation of EPDM [30]. Although there is a slight increase in the carbonyl group band over the entire aging period, Figure 4 confirms the absence of significant main-chain unsaturation loss under the mild aqueous conditions investigated, consistent with the EDX findings discussed above. In contrast, spectral changes are observed at higher wavenumbers (~2900 cm^−1^), reflecting molecular rearrangements and modifications in chain mobility induced by water uptake [36,37]. The absorption bands at approximately 1460 cm^−1^ and 1380 cm^−1^ are assigned, respectively, to the bending modes of –CH_2_– and –CH_3_ groups. Figure 4 shows a progressive shift towards lower frequencies with increasing immersion time, accompanied by a progressive attenuation. The shift in the CH_2_ and CH_3_ bands (the bond softening effect) and their broadening (the wider distribution of conformational environments) are consistent with a plasticization mechanism in which absorbed water disrupts secondary intermolecular interactions, increases free volume, and enhances local chain mobility within the amorphous regions of the network, without inducing covalent bond scission in the saturated backbone. The decrease in their intensity can be explained by the partial dissolution or leaching of chain segments, and/or oxidation or chain cleavage reactions on side groups. These two effects can reduce the local content of CH_2_ and CH_3_. Similarly, the bands at approximately 2920 cm^−1^ and 2850 cm^−1^, corresponding to the asymmetric and symmetric stretching modes of –CH_2_– groups, decrease in intensity.

### 3.3. Thermal Stability

**Figure 5:**
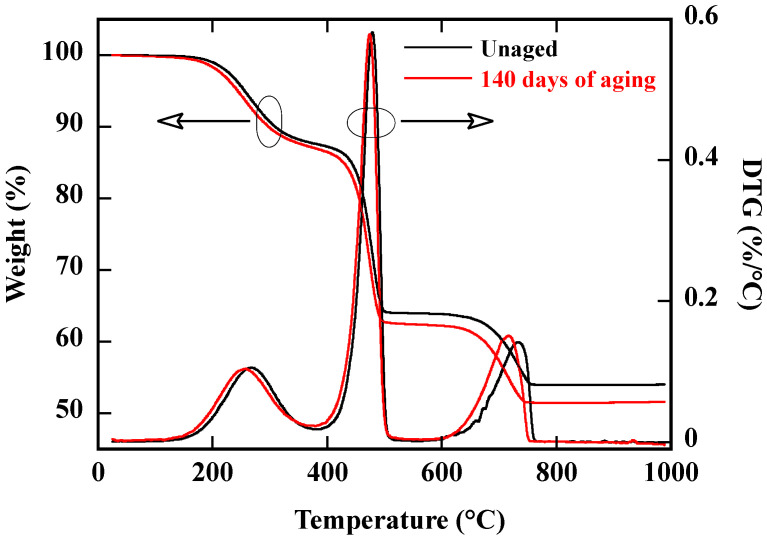
TGA-DTG spectra of unaged and 140-day-aged EPDM samples.

TGA analysis under nitrogen was chosen to characterize the material’s intrinsic thermal decomposition behavior and to isolate the effect of water-induced aging on the stability of the polymer backbone independently of any oxidative contribution. The thermal stability of unaged and aged EPDM specimens (140 days of immersion) was evaluated by simultaneous TGA/DTG analysis. The thermogravimetric profiles are presented in Figure 5 and exhibit a well-defined multi-step mass loss sequence reflecting the complex composition of the filled compound. No mass loss is recorded below 115 °C, confirming the absence of residual moisture or volatile contaminants in the tested specimens. The first degradation event, occurring between 115 °C and 380 °C with a DTG peak at approximately 260 °C, corresponds to a mass loss of 12–12.5% and is attributed to the volatilization and thermal decomposition of oils, plasticizers, and other low-molecular-weight non-rubber ingredients [38,39]. The second stage, spanning 370–500 °C with a DTG peak near 475–480 °C, involves a mass loss of 23.5–25% and is ascribed to main-chain scission of the EPDM backbone, a temperature range consistent with previously reported thermal degradation profiles for crosslinked EPDM compounds, which generally exhibit greater thermal stability than unsaturated diene rubbers [7]. A third degradation event is observed between 500 °C and 760 °C, with a DTG peak near 725 °C and a mass loss of 10–12%, attributable to the thermal decomposition of calcium carbonate (CaCO_3_) filler, in agreement with published data for EPDM/CaCO_3_ systems [38]. The residual mass above 760 °C, accounting for 50–54% of the initial specimen mass, corresponds to thermally stable inorganic components and incombustible mineral phases. Comparison of aged and unaged thermograms reveals only marginal shifts in DTG peak temperatures, with no new degradation events introduced by immersion. This demonstrates that 140 days of water immersion at ambient temperature does not alter the intrinsic thermal decomposition behavior of the EPDM compound in any significant manner, consistent with previous reports confirming the retention of thermal stability under moderate aging conditions and the onset of meaningful degradation only under intense thermal or oxidative exposure [7,38]. The near-total absence of difference between the aged and non-aged samples under nitrogen is physically consistent with the known thermal stability of EPDM and confirms that immersion in water does not alter the covalent structure of the polymer backbone.

### 3.4. Water Absorption and Surface Wettability

**Figure 6:**
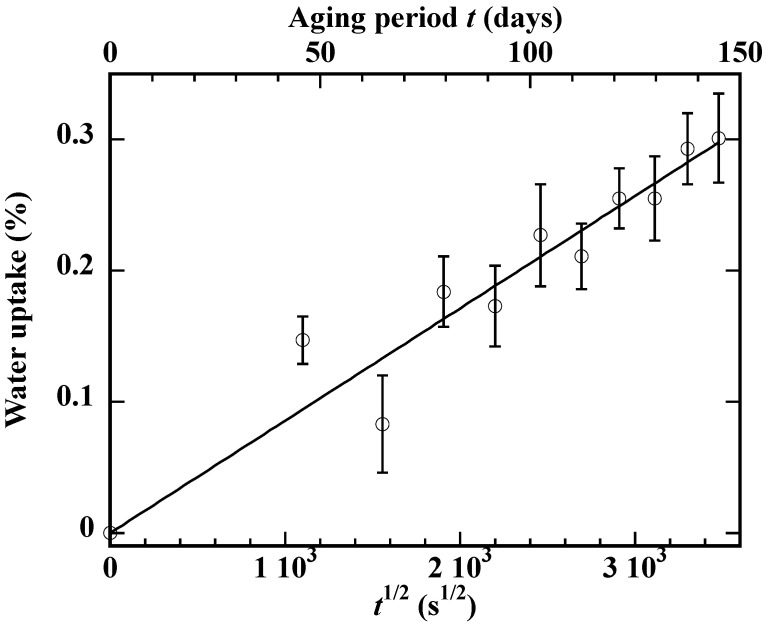
Water absorption over the aging time. The error bars represent the standard deviation. The solid curve is a Fickian trend of Equation (4).

Despite its saturated hydrocarbon backbone and inherently hydrophobic character, EPDM can absorb limited amounts of water through its microscopic free-volume regions and along polymer–filler interfaces, particularly under prolonged immersion. The diffusion of liquid water through EPDM has received relatively little attention in the recent literature, likely owing to the very low absorption levels involved [29]. Nevertheless, accurate characterization of water uptake kinetics is essential for understanding the progressive physicochemical changes documented in the preceding sections. The mass gain ΔM(*t*) as a function of immersion time is presented in Figure 6. Throughout the 140-day test period, the material exhibited only minimal water uptake, reaching approximately 0.30 wt%, a value that confirms the excellent hydrolytic resistance of EPDM at ambient temperature and is consistent with previously reported absorption levels for crosslinked EPDM compounds [40]. No clear saturation plateau was reached within the studied period, suggesting that equilibrium had not been fully attained after 140 days.

Fickian diffusion of water from a constant source, across the surface (*x* = 0) of a semi-infinite medium, results in a diffusion profile, at any time *t*, described by:

(1) $$C\left(x,t\right)=C_{S}\mathrm{erfc}\left(\frac{x}{2\sqrt{Dt}}\right)$$

C_S_ is the concentration of water on the surface of the medium. e and D the water diffusivity in the medium. The amount of water ΔM(*t*) absorbed by both sides of a sample of thickness λ, at time *t*, is given by the following equation:

(2) $$\Delta M\left(t\right)=2\int_{0}^{\infty }C_{S}\mathrm{erfc}\left(\frac{x}{2\sqrt{Dt}}\right)dx$$

After a sufficiently long time (*t* ⟶ ∞), when uniform saturation is reached, the amount of water absorbed by the sample will be:

(3) $$\Delta M\left(\infty \right)=C_{S}\ell$$

The expression (2) leads to the following result:

(4) $$\frac{\Delta M\left(t\right)}{\Delta M\left(\infty \right)}=\frac{4}{\ell }\sqrt{\frac{D\;t}{\pi }}$$

As shown in Figure 6, the experimental absorption kinetics can be well described by a classical Fick diffusion model, with a more pronounced increase occurring during the first ~30 days. However, Figure 6 clearly shows that the maximum immersion period of 140 days is insufficient for the EPDM to reach equilibrium with regard to absorbed water. Taking the equilibrium water uptake values between 0.4% and 0.5%, as cited in the literature [40], fitting Equation (4) to the experimental data yields a diffusion coefficient D ≈ 1 − 2 × 10^−10^ cm^2^.s^−1^, in good agreement with values reported for moisture diffusion in polymers [41,42]. The Fickian behavior is consistent with a mechanism governed primarily by molecular diffusion through the amorphous polymer network, modulated by the gradual relaxation of elastomeric chains. Over longer timescales, however, deviations from ideal Fickian kinetics may arise [20,21] as water molecules penetrating through microscopic pores interact chemically with hydrophilic groups within the matrix, converting free water into bound water and locally increasing the matrix’s affinity for moisture [43]. This effect appears to be limited, given the predominantly hydrophobic nature of EPDM. This could explain the absence of a clear saturation plateau (Figure 6) and the gradual changes in surface composition revealed by EDX and ATR-FTIR analyses (Figure 3 and Figure 4).

**Figure 7:**
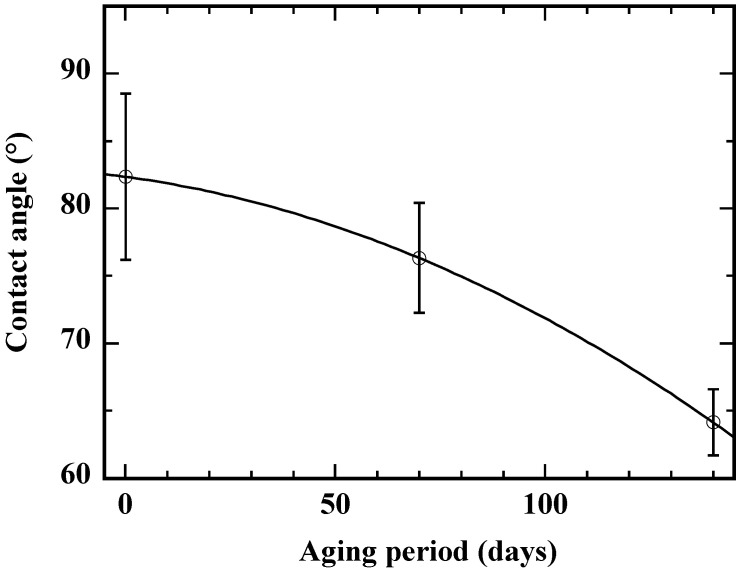
Contact angle of unaged and aged (70 and 140 days) EPDM samples. The error bars represent the standard deviation.

The evolution of surface wettability was assessed by static water contact angle measurements, with results shown in Figure 7. In the unaged state, the contact angle of 82.35° confirms the hydrophobic character of the EPDM surface. After 140 days of immersion, the contact angle decreased to 64.15°, indicating a progressive increase in surface hydrophilicity. The temporal evolution of the contact angle exhibits two distinct regimes: a modest decline during the first 70 days, followed by a more pronounced reduction between 70 and 140 days. This accelerating trend is consistent with the surface morphological and compositional changes described in Section 3.1 and Section 3.2, and can be attributed to several concurrent mechanisms. First, the leaching of low-molecular-weight hydrophobic species, such as plasticizers and processing oils, progressively exposes polar sites, residual catalytic species, and oxidized chain segments at the surface, increasing surface polarity [44]. Second, absorbed water induces localized plasticization of the near-surface polymer chains, reducing chain stiffness and facilitating the reorientation of buried polar functional groups towards the polymer–air interface [45]. Third, the volumetric swelling associated with water uptake modifies the microscale surface topography, as evidenced by the SEM observations in Figure 1. Consequently, increased surface roughness amplifies the intrinsic wetting behavior of the surface, further lowering the apparent contact angle in accordance with the Wenzel model. Finally, the conversion of free water into bound water, through interaction with hydrophilic groups within the matrix, generates localized polar regions that further contribute to enhanced wettability [43]. Collectively, these results demonstrate that water immersion induces a progressive and irreversible increase in surface polarity, driven by a combination of leaching, plasticization, surface reorganization, and topographical changes. Contact angle measurements thus provide a sensitive and integrative indicator of surface aging in filled EPDM systems, complementing the chemical and morphological characterization presented in the preceding sections [44,45].

### 3.5. Functional Properties

The effects of prolonged water immersion on the mechanical and electrical properties of EPDM were systematically tracked over 140 days.

#### 3.5.1. Mechanical Properties

**Figure 8:**
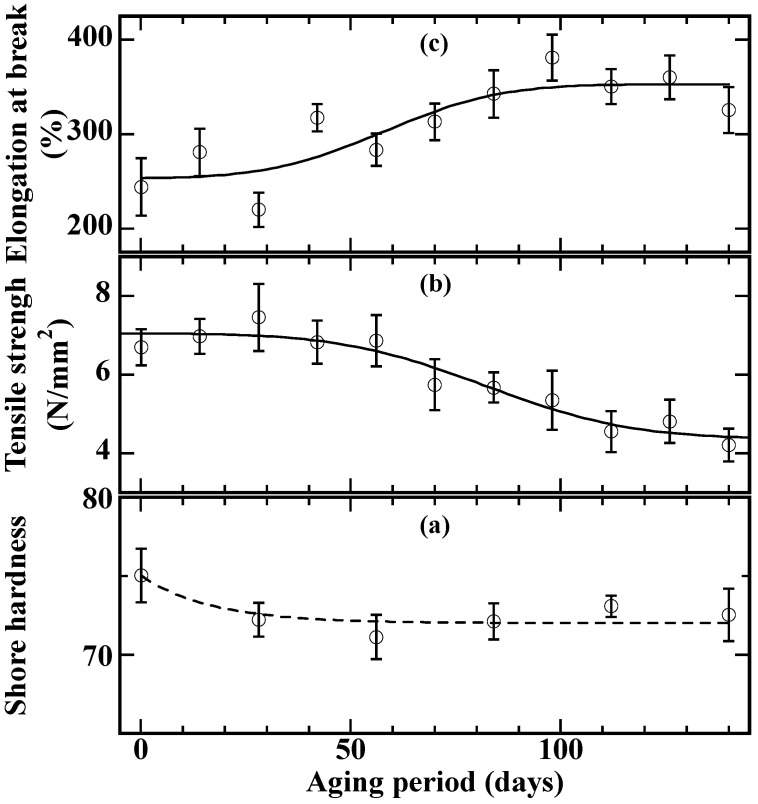
(**a**–**c**) Evolution of the mechanical properties over the aging time. The error bars represent the standard deviation. The solid curves are fitted with Equation (5) and the dashed curves with Equation (6).

Figure 8 illustrates the effects of prolonged immersion in water on filled EPDM samples:

(i) A slight drop in Shore A hardness units from 75 to 73 within the first 30 days of immersion, followed by a plateau (Figure 8a).
(ii) The tensile strength drops from approximately 7 N/mm^2^ to around 4.5 N/mm^2^ over 100 days, representing a loss of around 35% (Figure 8b).
(iii) A gradual increase in elongation at break, rising from around 230% to almost 390% over 100 days, an increase of approximately 70% (Figure 8c).

These results are the consequence of changes caused by water diffusion within the material over time, such as:

- water-induced plasticization due to an increase in free volume and chain mobility;
- weakening or even breaking of the physical bonds between the filler (particularly for hydrophilic silica fillers) and the matrix; and
- leaching of the vulcanization accelerators.

The first two effects observed are the mechanical properties most frequently encountered during the water-induced aging of filled elastomers [46]. Regarding elongation at break, the most frequently reported trend in the literature for EPDM aged in aqueous or thermal environments is a decrease over time. An increase in elongation at break following immersion in water is primarily observed in more hydrophilic elastomers, such as polyurethane and certain composites [47], where water-induced plasticization is very pronounced. It is also observed in EPDM immersed in oils or liquid plasticizers, where swelling reduces the effective cross-linking density and increases elongation at break [48]. The initial increase in elongation at break up to around 100 days could be an indication of enhanced ductility, caused by water-induced plasticization and stress relaxation. In summary, Figure 8 illustrates two distinct kinetic regimes in terms of mechanical properties. These regimes result from the successive stages of water penetration and interfacial degradation, which are described in the preceding sections. Hardness primarily depends on the matrix’s compressibility modulus, which reacts rapidly to plasticization caused by water absorbed during the first 30 days. Elongation at break, on the other hand, is governed by the integrity of the filler/matrix interface and by resistance to crack initiation and propagation. Both of these depend on the gradual accumulation of interfacial damage over longer timescales.

#### 3.5.2. Electrical and Dielectric Properties

**Figure 9:**
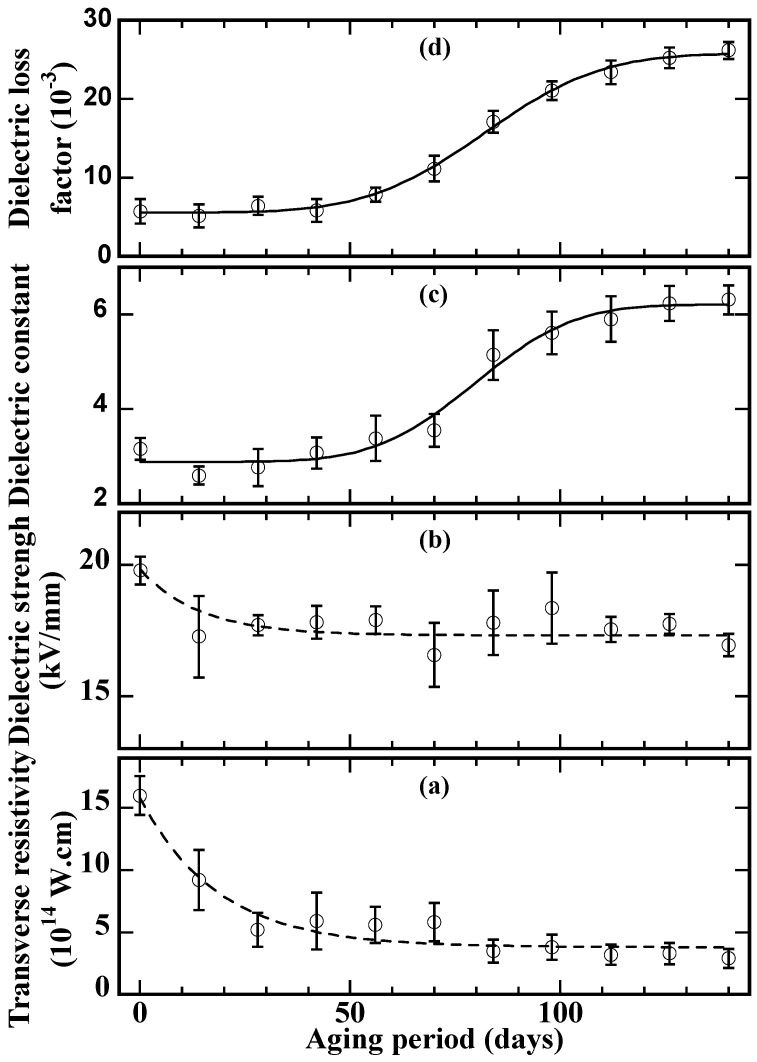
(**a**–**d**) Evolution of the electrical and dielectric properties over the aging time. The error bars represent the standard deviation. The solid curves are fitted with Equation (5) and the dashed curves with Equation (6).

As can be seen in Figure 9, the results of the electrical measurements show that aging in an aqueous environment significantly influences the properties of filled EPDM. As with the mechanical properties, this appears to be due to two successive and mechanistically distinct stages: an initial, diffusion-driven deterioration in resistivity and dielectric strength, followed by a slower but substantial increase in the dielectric constant and dielectric loss factor, caused by interfacial polarization and increased dipole mobility. More specifically, we find that:

(i) Volume resistivity and dielectric strength both undergo rapid and significant deterioration during the first 30 days of immersion. Resistivity drops to approximately two-thirds of its initial value, while dielectric strength decreases from around 20 kV/mm to 16–16.5 kV/mm, representing a total reduction of 15% to 20%.
(ii) The relative permittivity and dielectric loss factor demonstrate a gradual, step-like change. For about thirty days, both parameters remain approximately stable, suggesting that the initial penetration of water is insufficient to significantly perturb the bulk dielectric response, and that early network relaxation or leaching of low-molecular-weight additives may partially compensate for moisture-induced polarization effects. Between approximately 70 and 100 days, both parameters increase sharply before approaching a plateau near 120–140 days.

We attribute the rapid decline in resistivity (Figure 9a) and dielectric strength (Figure 9b) to the initial water molecules, which occupy accessible polar sites such as residual functional groups, charge/matrix interfaces and microvoids. This ‘bound water’, which is strongly adsorbed, forms a network of percolating conductive pathways that governs the observed drop in resistivity and dielectric strength during the first 30 days. Beyond this point, any additional water absorption would essentially correspond to ‘free water’ diffusing into already isolated micropores without creating new conduction pathways. The conductive network is then topologically saturated (in the sense of percolation theory) and, consequently, the marginal addition of water to sites already disconnected from the main conductive path does not worsen the electrical properties. However, we cannot rule out the contribution of slight structural reorganization (e.g., partial recrystallisation or rearrangement of charge-carrying chains at the surface), which may also stabilize the interfaces and prevent the formation of new conductive pathways.

The increase in dielectric permittivity and dielectric loss factor observed after around 70 days is more closely related to the formation of sufficiently developed water-polymer-charge interfaces that allow for the accumulation of free charges, rather than to the amount of water absorbed directly. This interfacial polarization mechanism involves the migration of ionic charge carriers (resulting from the partial dissolution of ionic species or the dissociation of impurities) towards charge/matrix or water/polymer interfaces, where these charges accumulate to create dipoles. This mechanism requires the ionic diffusion paths through the polymer to be sufficiently well-established (a process that takes longer than simple water absorption via Fick’s molecular diffusion) and the concentration of mobile ionic species to reach a sufficient threshold. The gradual nature of ionic diffusion through a tortuous polymer network could, in this case, result in a dielectric response that is delayed by several weeks.

The early rapid decline in resistivity and dielectric strength is consistent with a rapid diffusion-controlled stage in which water penetrates the amorphous regions and free-volume elements of the polymer network, inducing plasticization, reducing intermolecular cohesion, and increasing charge carrier mobility through hydrogen-bonded water clusters and microvoid pathways [43]. In conductive-filled EPDM, water preferentially accumulates at filler/matrix interfaces, weakening interfacial adhesion, promoting partial debonding, reducing tunneling distances between conductive filler particles and, thereby, enhancing percolative charge transport [49]. Moreover, any associated local electric field distortions will further reduce the breakdown strength of the material [43]. Beyond about 30 days, both properties stabilize near their reduced values as the moisture concentration gradient diminishes and the network approaches a quasi-equilibrium conductive state, as observed in hydrothermally aged polyolefin-based insulating materials [50].

Accumulated water promotes the formation of microvoids and intensifies the local field, creating preferential initiation sites for electrical breakdown [43]. The high intrinsic permittivity of water increases the material’s overall dielectric constant after several weeks of water absorption due to contributions related to orientational and interfacial polarization [37]. Furthermore, the increased dipole mobility resulting from the gradual absorption of water increases the dielectric loss factor. These effects are typical of polymers that have been subjected to moisture absorption over a long period [51]. Given the well-established hydrolytic stability of EPDM’s saturated backbone, the observed stabilization beyond 100 days may reflect the progressive weakening of the interfaces between the filler and the matrix.

### 3.6. Time Evolution of Properties: An Attempt at Interpretation

As shown in Figure 8 and Figure 9, monitoring the various functional properties during prolonged immersion in water at constant temperature reveals two distinct regimes of change that reflect the successive physicochemical mechanisms discussed in the preceding sections. The first involves a rapid decline in Shore hardness (Figure 8a), transverse resistivity (Figure 9a) and dielectric strength (Figure 9b) occurring over a timescale of 20 to 30 days. The second consists of a slower, gradual evolution, exhibiting a step-like character, in tensile strength (Figure 8b), elongation at break (Figure 8c), dielectric constant (Figure 9c) and dielectric loss factor (Figure 9d), unfolding over several tens of days.

Within the bounds of the aging period examined and the sampling intervals employed, we sought to quantify these two regimes through appropriate analytical expressions. Properties undergoing an abrupt, step-like transition are described by Equation (5):

(5) $$Y\left(t\right)=Y\left(0\right)+\frac{Y\left(\infty \right)-Y\left(0\right)}{2}\left[1+erf\left(\frac{t-t_{0}}{\tau }\right)\right]$$

where *t*_0_ denotes the time at which the transition occurs and *τ* denotes its characteristic duration. Properties that declined rapidly from the outset of immersion (*t*_0_ = 0) are described by Equation (6):

(6) $$Y\left(t\right)=\begin{array}{c} Y\left(0\right)\\ Y\left(\infty \right)+\left[Y\left(0\right)-Y\left(\infty \right)\right]\exp\left(-\frac{t-t_{0}}{\tau }\right) \end{array}\begin{array}{c} t<t_{0}\\ t\geq t_{0} \end{array}$$

The solid and dashed curves in Figure 8 and Figure 9 represent the fits obtained using Equations (5) and (6), respectively. The values of *t*_0_ and *τ* extracted for each property are compiled in Table 1, as well as the values of the correlation coefficient R for all the functional property fits, to give the reader insight into the reliability of the results.

The disparity in the experimental measurements of elongation at break (see Figure 8c) results in less precise values for t_0_ and τ. This is confirmed by a correlation coefficient R that is somewhat distant from 1. Several factors contribute to the high level of uncertainty observed in certain mechanical properties, particularly elongation at break.

- Filled elastomers naturally exhibit significant variation in this parameter, which is highly sensitive to local defects (e.g., microporosity, filler agglomerates and crosslinking heterogeneities) in both unaged and aged specimens.
- Since water absorption is a diffusion process, aged samples exhibit a water content gradient between the surface and the core, particularly at short aging times. This leads to heterogeneity in mechanical properties along the specimen and increases the disparity in results.
- The limited number of specimens (five) increases statistical uncertainty.

The trend shown by Table 1, across all the analyzed functional properties, allows us to distinguish two behavioral families:

(a) Properties with immediate degradation (*t*_0_ = 0):

These three properties (Shore hardness, transverse resistivity and dielectric strength) begin degrading from the very start of immersion, with very similar characteristic times *τ* (~14–18 days). This points to a common, rapid mechanism, most likely the penetration of free water into the material. Water infiltrates pores or interfaces and immediately disrupts: the surface mechanical response (Shore hardness, a surface-sensitive property), electrical conduction pathways (transverse resistivity) and breakdown resistance (dielectric strength). The consistency of *τ* across these three quantities is a strong argument for a single diffusion-driven mechanism.

(b) Properties with delayed degradation (*t*_0_ ≠ 0):

These properties only degrade after an incubation period of about thirty days, pointing to a slower mechanism, most likely a structural modification of the polymer network: progressive plasticization, hydrolysis of chemical bonds, or delayed swelling affecting the cohesion of the EPDM network. There are two notable observations: (i) The *t*_0_ value of 58 days for elongation at break is slightly lower than that for tensile strength and dielectric properties (80–82 days). (ii) The uncertainty of both *t*_0_ (±14 days) and *τ* (±27 days) is very high, making these values unreliable. By contrast, the other properties (tensile strength, dielectric constant and dielectric loss factor) exhibit highly similar *t*_0_ and *τ* values and extremely low uncertainty. These results suggest an underlying mechanism and demonstrate the excellent reproducibility of the measurements.

## 4. Conclusions

The following scenario explicitly highlights the connections between the morphological, microstructural and surface characterization results, and the chronology of changes in functional properties:

- Day 0–Day 30: rapid diffusion of water into the amorphous regions and at the filler/matrix interfaces → formation of a percolating network of conductive pathways → rapid decrease in resistivity, dielectric, dielectric strength and hardness. Water uptake induces volumetric swelling and alters the microscale surface topography, causing the surface to roughen gradually and develop microcavities and microcracks and amplifies the intrinsic wetting behavior of the material → the contact angle decreases (hydrophilization) and the tensile modulus begins to decrease (plasticization).
- Day 30–Day 80: slowed water absorption (diffusion into denser regions) → stabilized electrical properties (saturated conductive network). FTIR shows a gradual decrease in the CH_2_/CH_3_ bands, which is consistent with the leaching of low-molecular-weight components.
- Day 80–Day 140: Gradual accumulation of ionic species at the filler/matrix interfaces → activation of polarization → increase in dielectric constant and dielectric loss factor. Enrichment in Ca, Al and Si is detected at the surface by EDX, which is consistent with the gradual erosion of the matrix and exposure of the fillers. Concurrently, oxygen-containing species produced during chemical degradation may recombine, partially restoring the damaged network through delayed oxidative crosslinking, a process that limits the degradation of certain functional properties over an extended period [52].

This clear separation into two distinct temporal regimes is particularly valuable for aging modelling and for defining end-of-life criteria depending on the intended application, whether the primary concern is electrical performance or mechanical integrity. These findings underscore the importance of accounting for long-term ambient moisture exposure in the design and reliability assessment of EPDM-based cable insulation systems.

## Figures and Tables

**Table 1:** Values of t_0_, τ and the correlation coefficient R obtained from the fits in Figure 8 and Figure 9 using Equations (5) and (6).

Property	*t*_0_ (Days)	*τ* (Days)	R
Shore hardness	0	17 ± 2	0.99943
Transverse resistivity	0	18 ± 4	0.97056
Dielectric strength	0	14 ± 7	0.85051
Tensile strength	82 ± 8	39 ± 16	0.96601
Elongation at break	58 ± 14	31 ± 27	0.86179
Dielectric loss factor	82 ± 2	31 ± 3	0.99853
Dielectric constant	80 ± 4	26 ± 6	0.9896

## Data Availability

The original contributions presented in this study are included in the article. Further inquiries can be directed to the corresponding authors.
